# Loss of primary cilia promotes mitochondria-dependent apoptosis in thyroid cancer

**DOI:** 10.1038/s41598-021-83418-3

**Published:** 2021-02-18

**Authors:** Junguee Lee, Ki Cheol Park, Hae Joung Sul, Hyun Jung Hong, Kun-Ho Kim, Jukka Kero, Minho Shong

**Affiliations:** 1 Department of Pathology, Daejeon St. Mary’s Hospital, College of Medicine, The Catholic University of Korea, Seoul, 06591 Republic of Korea; 2 Clinical Research Institute, Daejeon St. Mary’s Hospital, College of Medicine, The Catholic University of Korea, Daejeon, 34943 Republic of Korea; 3 Research Center for Endocrine and Metabolic Diseases, Chungnam National University School of Medicine, Daejeon, 35015 Republic of Korea; 4 Department of Nuclear Medicine, Chungnam National University Hospital and College of Medicine, Daejeon, 35015 Republic of Korea; 5 Research Centre for Integrative Physiology and Pharmacology, Institute of Biomedicine, University of Turku, Kiinamyllynkatu 10, 20520 Turku, Finland; 6 Department of Internal Medicine, Chungnam National University School of Medicine, 266 Munhwaro, Daejeon, 35015 Republic of Korea

**Keywords:** Cancer, Cell biology, Endocrinology, Oncology

## Abstract

The primary cilium is well-preserved in human differentiated thyroid cancers such as papillary and follicular carcinoma. Specific thyroid cancers such as Hürthle cell carcinoma, oncocytic variant of papillary thyroid carcinoma (PTC), and PTC with Hashimoto’s thyroiditis show reduced biogenesis of primary cilia; these cancers are often associated the abnormalities in mitochondrial function. Here, we examined the association between primary cilia and the mitochondria-dependent apoptosis pathway. *Tg-Cre;Ift88*^flox/flox^ mice (in which thyroid follicles lacked primary cilia) showed irregularly dilated follicles and increased apoptosis of thyrocytes. Defective ciliogenesis caused by deleting the *IFT88* and *KIF3A* genes from thyroid cancer cell lines increased VDAC1 oligomerization following VDAC1 overexpression, thereby facilitating upregulation of mitochondria-dependent apoptosis. Furthermore, VDAC1 localized with the basal bodies of primary cilia in thyroid cancer cells. These results demonstrate that loss-of-function of primary cilia results in apoptogenic stimuli, which are responsible for mitochondrial-dependent apoptotic cell death in differentiated thyroid cancers. Therefore, regulating primary ciliogenesis might be a therapeutic approach to targeting differentiated thyroid cancers.

## Introduction

The primary cilium is a non-motile, microtubule-based sensory organelle that receives mechanical and chemical stimuli from the environment and transduces external signals into the cell^1^. The tips of primary cilia, which are present in the apical membrane of thyroid follicular cells (thyrocytes), face into the follicular lumen^2^. The primary cilia of murine thyroid follicular cells play a role in maintaining globular follicle structures by acting on cell polarity^3^. Loss-of-function (LOF) of primary cilia in murine thyroid follicles results in abnormal and irregular follicles that eventually develop into papillary and solid proliferative nodules^3^.

The primary cilium is well-preserved in human differentiated thyroid cancers, including papillary and follicular carcinoma, and their frequency and length appear similar to those of normal thyroid follicles^2^. Interestingly, the frequency of ciliated thyroid cancer cells is markedly lower in Hürthle cell carcinoma, oncocytic variant of papillary carcinoma (PTCov), and PTC with Hashimoto’s thyroiditis (PTC-HT), which are usually associated with mitochondrial dysfunction^2^. However, we do not know whether ciliogenesis is linked with mitochondrial function in thyroid cancer cells.

Mitochondria are crucial regulators of cell death through a process called the mitochondria-dependent (intrinsic) pathway of apoptosis. Typically, mitochondrial outer membrane permeabilization (MOMP) is responsible for mediating the intrinsic apoptotic pathway. The voltage-dependent anion channel (VDAC), a component of MOMP, participates in mitochondria-dependent apoptosis by promoting cytochrome *c* release^4,5^. VDAC oligomerization, followed by VDAC overexpression, may represent a common mechanism by which various apoptogens act through different initiating cascades^6^. Moreover, VDAC function extends beyond the mitochondria, and VDACs localize to the basal body of the primary cilium, where VDAC1 and VDAC3 negatively regulate ciliogenesis^7^. Recent reports show that dysfunction of primary cilia increases apoptotic cell death in glioblastoma, or induce neuron apoptosis in mice^8,9^. However, the relationship between primary cilia and cell death via activation of the mitochondrial apoptotic pathway is unclear.

Here we established a mouse model with thyrocyte-specific loss of primary cilia (*Tg-Cre;Ift88*^flox/flox^) and human thyroid cancer cell lines with ciliary loss by silencing the *KIF3A* or *IFT88* gene. To identify the role of ciliogenesis with respect to the viability of normal thyrocytes and thyroid cancer cells, we examined apoptotic cell death in murine thyroid follicular cells and human thyroid cancer cells devoid of primary cilia. We found that mice lacking primary cilia in thyroid follicular cells showed upregulated apoptotic cell death, resulting in altered follicular structure, and that inhibiting ciliogenesis in thyroid cancer cell lines resulted in VDAC1 oligomerization following VDAC1 overexpression, leading ultimately to apoptosis. Additionally, we demonstrate that VDAC1 is localized to the primary cilia of thyroid follicular cells. Taken together, these results establish that LOF of primary cilia is a novel apoptogenic stimulus in thyroid cancers. Therefore, inhibiting primary cilia might be a therapeutic target for thyroid cancers.

## Results

### Murine thyroid devoid of primary cilia after inactivation of the Ift88 gene shows altered follicular structure

Assembly and maintenance of primary cilia are dependent on a transport system controlled by intraflagella transport (IFT) family proteins^10^. Knockout of IFT88, an IFT retrograde complex B subunit, in murine thyroid follicles prevents ciliogenesis^3^. To study the effect of thyrocyte-specific deletion of the *Ift88* gene, we used mice expressing Cre recombinase under the control of the thyroglobulin (Tg) promoter. *Tg-Cre* is constitutively active from embryonic day 14.5^11,12^. These Tg-Cre-expressing mice were crossed with *Ift88*^flox/flox^ mice to generate *Tg-Cre;Ift88* floxed mice that exhibit thyroid follicle-specific ciliary loss.

**Figure 1:**
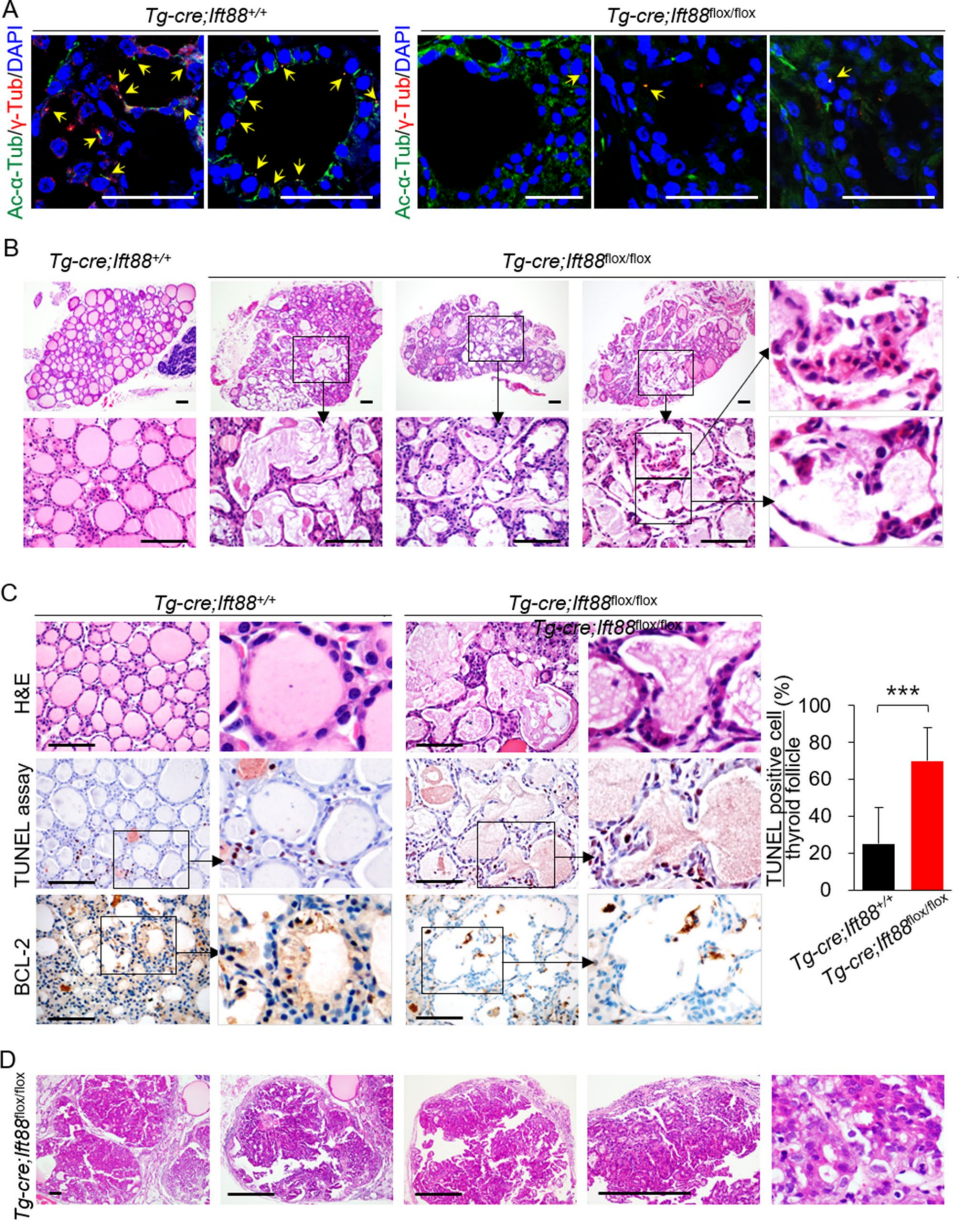
Loss of function of *Ift88*-mediated resulted in ciliary loss and increased apoptosis. (**A**) Immunofluorescence images showing primary cilia in thyroid of *Tg-Cre;Ift88*^+/+^ and *Tg-Cre;Ift88*^flox/flox^ mice. Primary cilia were confirmed by staining with anti-acetylated α-tubulin (Ac-α-Tub, green) and anti-γ-tubulin (γ-Tub, red) antibodies. Primary cilia are indicated by arrows. Scale bar, 10 μm. (**B**) The thyroid of *Tg-Cre;Ift88*^flox/flox^ mice (aged 14 weeks) shows irregular dilated follicles with a flat epithelium and luminal colloid depletion. Scale bar, 10 μm. (**C**) *Tg-Cre;Ift88*^flox/flox^ mice (aged 14 weeks) showed irregularly dilated thyroid follicles comprising shrunken eosinophilic cells with compact nuclei (H&E). Scale bar, 10 μm. The number of TUNEL-positive follicular cells per follicle in the wild-type control and *Tg-Cre;Ift88*^flox/flox^ mice was 25 ± 20% and 70 ± 18%, respectively (*P* < 0.0001). BCL-2-positive follicular cells were rarely observed in the irregularly dilated thyroid follicles of *Tg-Cre;Ift88*^flox/flox^ mice. ***P < 0.001. (**D**) In 35 week-old *Tg-Cre;Ift88*^flox/flox^ mice, the dilated follicles developed into solid proliferative thyroid nodules. Scale bar, 10 μm.

Immunofluorescence analysis of primary cilia markers acetylated α-tubulin and γ-tubulin confirmed that thyroid follicular cells in *Tg-Cre;Ift88*^+/+^ mice had primary cilia (Fig. 1A). By contrast, primary cilia were rarely detected on thyroid follicles in *Tg-Cre;Ift88*^flox/flox^ mice (Fig. 1A). The thyroids of 7 week-old *Tg-Cre;Ift88*^flox/flox^ mice exhibited irregularly dilated follicles with colloid depletion. These dilated follicles comprised shrunken, hypereosinophilic cells with compact nuclei, which were morphologically compatible with apoptosis (Fig. 1B).

Terminal deoxynucleotidyl transferase dUTP nick-end labeling (TUNEL) assays revealed a higher proportion of apoptotic follicular cells within the irregularly dilated thyroid follicles of 7 week-old *Tg-Cre;Ift88*^flox/flox^ mice than in those of wild-type control mice (Fig. 1C). In addition, thyroid follicular cells in *Tg-Cre;Ift88*^flox/flox^ mice showed lower expression of anti-apoptotic BCL-2 protein than those from control mice (Fig. 1C). Therefore, the thyroid follicles of 7 week-old *Tg-Cre;Ift88*^flox/flox^ mice show increased apoptosis.

The irregularly dilated follicles which increased with apoptosis eventually developed into papillary and solid proliferative follicular nodules in the thyroids of 35 week-old *Tg-Cre;Ift88*^flox/flox^ mice (Fig. 1D).

### LOF of primary cilia in thyroid cancer cell lines results in increased apoptosis

Next, we investigated whether loss of primary cilia induces apoptosis in thyroid cancer cell lines. Lactate dehydrogenase (LDH) levels have been used as an indicator of late apoptosis in various studies. TPC1 and BCPAP cell lines had lower LDH levels than those of other human thyroid carcinoma cell lines (PTC cell lines, TPC1 and BCPAP; anaplastic thyroid cancer cell lines, 8505C, Hth7 and SW1736; Hürthle cell carcinoma cell line, XTC.UC1) (Supplementary Fig. S1A) and had well-preserved primary cilia (similar to those of normal thyroid follicular cells) (Supplementary Fig. S1B)^2^, indicating an inverse correlation between the frequency of primary cilia and apoptosis. MTT cell viability assays revealed that TPC1 and BCPAP cells exhibited more cell death than 8505C and Hth7 cells after loss of primary cilia (Supplementary Fig. S1C). Based on these results, we selected TPC1 and BCPAP as the best cell lines to demonstrate that loss of primary cilia induces apoptosis in thyroid cancer cell lines.

**Figure 2:**
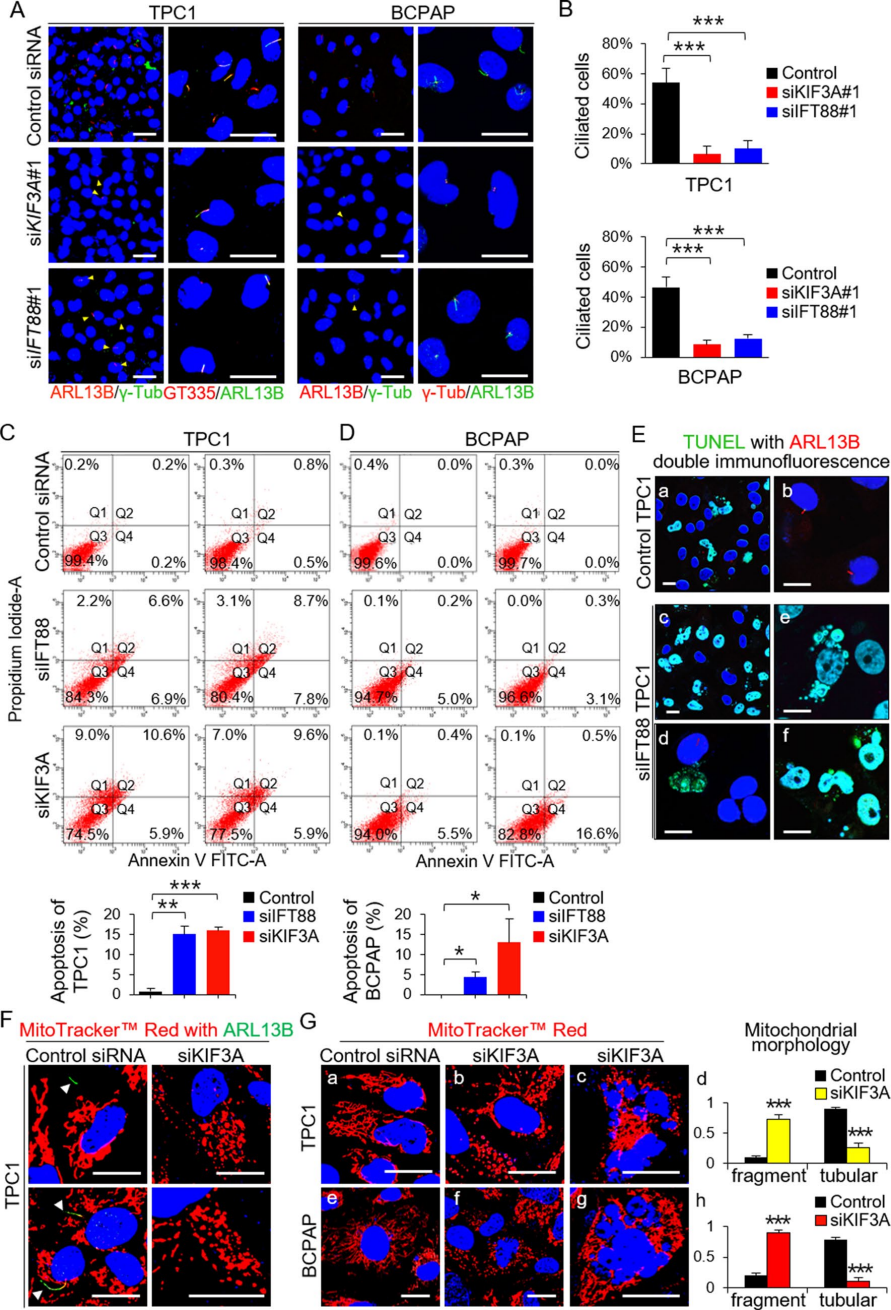
Loss-of-function of primary cilia increases apoptosis in thyroid cancer cell lines. (**A**) The number of cells with primary cilia were determined by immunofluorescence staining with antibodies specific for ARL13B (axoneme), anti-γ-tubulin (basal body), and GT335 (axonemes with basal bodies). Cell nuclei were stained with DAPI. Scale bar, 10 μm. (**B**) Frequency of ciliated cells in the *KIF3A*-KD, *IFT88*-KD, and negative control siRNA-transfected cell populations (TPC1 and BCPAP). *P < 0.05, **P < 0.01, ***P < 0.001, NS; not significant. (**C**, **D**) FITC-conjugated Annexin V and PI assay to measure apoptosis of *KIF3A*-deficient or *IFT88*-deficient TPC1 and BCPAP cells and negative control siRNA-transfected cells. Bar graphs show average percentage of apoptotic cells (Q2 + Q4). *P < 0.05, **P < 0.01, ***P < 0.001. (**E**) More *IFT88*-KD TPC1 cells than control TPC1 cells were TUNEL-positive (a, c). TUNEL with ARL13B double immunofluorescence staining revealed that TUNEL-positive *IFT88*-KD TPC1 cells (green) had no primary cilia (red). Scale bar, 10 μm. (**F**) Immunofluorescence images showing altered mitochondrial morphology and dynamics. The arrowheads indicate the primary cilia. Scale bar, 10 μm. (**G**) Immunofluorescence images showing signs of apoptosis in thyroid cancer cell lines with ciliary loss. These apoptotic cells showed highly fragmented mitochondria. Scale bar, 10 μm. ***P < 0.001.

The primary cilium was visualized by immunofluorescence staining with an anti-ARL13B antibody (which detect the axonemes), an anti-GT335 antibody (which detects axonemes with a basal body), and anti-γ-tubulin (which detects the basal body)(Fig. 2A). Primary cilia were detected in 54.05 ± 9.28% of TPC1 cells and in 46.54 ± 6.58% of BCPAP cells under serum starvation conditions (Fig. 2A and B). The kinesin family member 3A (*KIF3A*) and intraflagellar transport 88 (*IFT88*) genes encode important proteins involved in cilium biogenesis. Knockdown (KD) of *KIF3A* or *IFT88* by serum starvation resulted in significant decreases in the percentage of ciliated TPC1 (si*KIF3A* = 6.58 ± 5.54%; si*IFT88* = 10.14 ± 5.28%) and BCPAP (si*KIF3A* = 8.40 ± 2.61%; si*IFT88* = 12.40 ± 2.61%) cells (Fig. 2B). The efficiency of specific siRNA-mediated KD of *KIF3A* or *IFT88* in the PTC cell lines is shown in Supplementary Fig. S2. This finding suggests that ciliogenesis, a process regulated by KIF3A and IFT88, is preserved in thyroid cancer cells.

Next, we examined apoptotic cell death by performing Annexin V-FITC and PI staining and flow cytometry-based quantification. We found that KD of *KIF3A* or *IFT88* in thyroid cancer cell lines led to increased apoptotic cell death (Fig. 2C). Annexin V(+)/PI(+)(Q2) cells represent the late apoptotic population and Annexin V(+)/PI(-)(Q4) cells represent the early apoptotic population. TPC1 populations with defective *IFT88* or *KIF3A* harbored higher numbers of cells undergoing late apoptosis (si*IFT88* = 7.65 ± 1.48%, *p* = *0.011*; si*KIF3A* = 10.10 ± 0.71%, *p* = *0.002*) and early apoptosis (si*IFT88* = 7.35 ± 0.64%, *p* = *0.002*; si*KIF3A* = 5.90 ± 0.00%, *p* = *0.0003*) than TPC transfected with negative control siRNA late apoptosis = 0.50 ± 0.42%; early apoptosis = 0.35 ± 0.21%)(Fig. 2C). The number of *IFT88*-deficient or *KIF3A*-deficient BCPAP cells that exhibited early apoptosis was higher (si*IFT88* = 4.05 ± 1.34%, *p* = *0.025*; si*KIF3A* = 12.55 ± 5.72%, *p* = *0.045*) than that of cells transfected with negative control siRNA (Fig. 2D).

We then performed double detection of TUNEL and primary cilia in these cells to demonstrate that thyroid cancer cells lacking primary cilia are apoptotic. TUNEL-positive cancer cells within the *IFT88*-deficient TPC1 population showed loss of primary cilia (Fig. 2E-c, d). In particular, TUNEL-positive cells showing apoptotic nuclei lacked primary cilia (Fig. 2E-e, f).

It is widely accepted that mitochondrial fragmentation occurs during apoptosis^13,14^. Therefore, we used a confocal laser scanning microscope to examine mitochondrial morphology and the dynamics of *KIF3A*-deficient thyroid cancer cell lines and negative control siRNA-transfected cells stained with MitoTracker Red. TPC1 with primary cilia showed long, tubular mitochondrial networks, while TPC1 without primary cilia showed globular shaped mitochondria (Fig. 2F). Negative control siRNA-transfected TPC1 and BCPAP cells showed typical tubular mitochondria (Fig. 2G-a and G-e). By contrast, *KIF3A*-deficient TPC1 and *KIF3A*-deficient BCPAP cells showed small globular and ring-shaped mitochondria, which are indicative of increased fission and decreased fusion (Fig. 2G-b, d and G-f, h). More cells showing signs of apoptosis (i.e., nuclear fragmentation) were noted in thyroid cancer cell lines with ciliary loss than in thyroid cancer cells with primary cilia (Fig. 2G-c and G-g).

### LOF of primary cilia in thyroid cancer cell lines increases oligomerization of VDAC1

**Figure 3:**
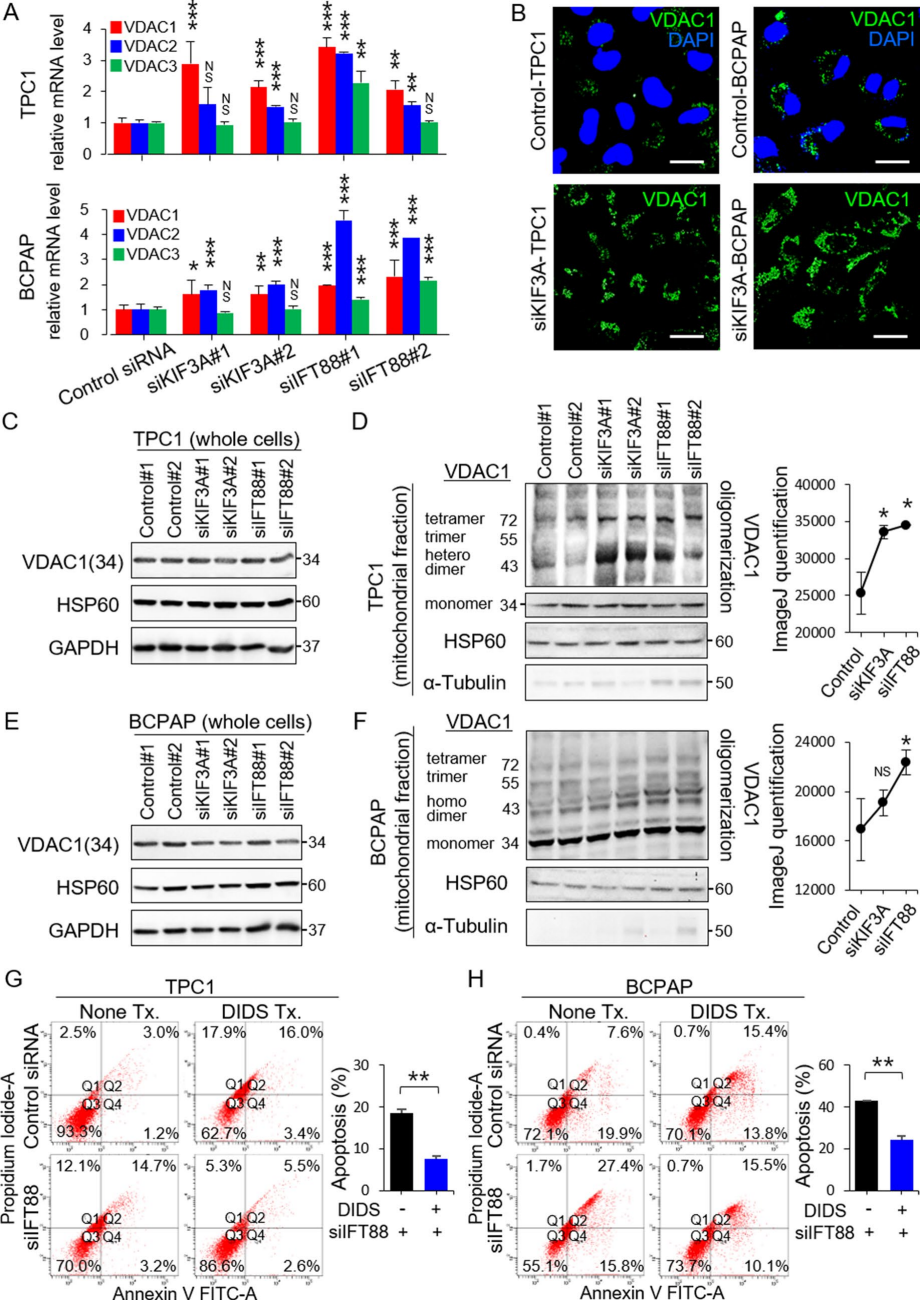
Loss-of-function of primary cilia in thyroid cancer cell lines upregulates the mitochondria-dependent apoptosis pathway. (**A**) Expression of *VDAC1*, *VDAC2*, and *VDAC3* mRNA in human thyroid cancer cells (TPC1 and BCPAP), with or without ciliary loss. **P < 0.01, ***P < 0.001, NS; not significant. (**B**) Comparison of immunofluorescence staining of VDAC1 between negative control siRNA-transfected cells and *KIF3A*-deficient TPC1 or BCPAP cells. Scale bar, 10 μm. (**C**) Western blot analysis of VDAC1 and HSP60 (a mitochondrial volume marker) expression in whole-cell lysates of *KIF3A*-KD or *IFT88*-KD TPC1 compared with that in negative control siRNA-transfected cells. (**D**) Analysis of the oligomeric status of VDAC1 in the mitochondrial fractions of *KIF3A*-KD and *IFT88*-KD TPC1 cells compared with negative control siRNA-transfected cells. A line graph was generated from the ImageJ data using arbitrary area units. *P < 0.05. (**E**) Western blot analysis of VDAC1 and HSP60 expression in whole-cell lysates of *KIF3A*-KD and *IFT88*-KD BCPAP cells compared with negative control siRNA-transfected cells. (**F**) Analysis of the oligomeric status of VDAC1 in the mitochondrial fraction of *KIF3A*-KD and *IFT88*-KD BCPAP cells compared with negative control siRNA-transfected cells. A line graph was generated from the ImageJ data using arbitrary area units. *P < 0.05, NS; not significant. (**G**, **H**) Flow cytometry analysis of apoptosis in *IFT88*-deficient TPC1/BCPAP cells treated with DIDS. The levels of apoptosis in *IFT88*-deficient TPC1 or BCPAP cells treated with DIDS were markedly less than those in untreated *IFT88*-KD cells. Bar graphs show average percentages of apoptotic cells (Q2 + Q4). **P < 0.01.

Apoptosis can be initiated by one of two pathways: the intrinsic (mitochondria-dependent) pathway or the extrinsic (death receptor-mediated) pathway^15^. VDAC1 plays a critical role in the mitochondria-associated apoptosis pathway^6^. VDAC1 overexpression induced by various apoptogenic stimuli causes oligomerization of mitochondrial VDAC1, leading to cell apoptosis^16,17^. To explore the role of primary cilia in mitochondria-associated apoptosis in thyroid carcinomas, we examined expression of *VDAC1*, *VDAC2*, and *VDAC3* mRNA in human PTC cells with or without ciliary loss. Expression of *VDAC1* and *VDAC2* mRNA was higher in *KIF3A*-deficient or *IFT88*-deficient TPC1 cells and BCPAP cells (Fig. 3A) than in the corresponding negative control siRNA-transfected cells. These results were supported by immunofluorescence staining, which revealed that *KIF3A*-deficient TPC1 and *KIF3A*-deficient BCPAP cells showed higher VDAC1 expression than the respective negative control siRNA-transfected cells (Fig. 3B).

Subsequently, we examined VDAC1 protein levels by western blot analysis. Increased expression of VDAC1 mRNA expression was not mirrored by increased expression of VDAC1 protein. We found no difference in the amount of mitochondria expressing HSP60 between thyroid cancer cells with or without primary cilia (Fig. 3C and E). Immunofluorescence staining revealed a clear difference in VDAC1 expression. Thus, we analyzed the oligomeric status of VDAC1 in *KIF3A*-deficient or *IFT88*-deficient thyroid cancer cell lines. Several distinct VDAC1 protein bands were identified by immunoblotting with anti-VDAC1 antibodies (Abcam ab15895 and ab14734), which corresponded to VDAC1 monomers, dimers, trimers, tetramers, and multimers. VDAC1 oligomerization increased significantly in *KIF3A*-deficient or *IFT88*-deficient TPC1 cells (si*KIF3A*, *p* = *0.030*; si*IFT88*, *p* = *0.023*) (Fig. 3D). VDAC1 oligomerization increased significantly in *KIF3A*-deficient or *IFT88*-deficient BCPAP, but not significantly in *KIF3A*-deficient BCPAP (si*KIF3A*, *p* = *0.188*; si*IFT88*, *p* = *0.050*)(Fig. 3F). Taken together, these results indicate that loss of primary cilia from thyroid cancer cells results in *VDAC1* overexpression, increased VDAC1 oligomerization, and upregulated apoptosis. Therefore, LOF of primary cilia in thyroid cancer cells acts as an apoptogenic stimulus for the mitochondria-dependent apoptosis pathway.

To support our conclusion that VDAC1 mediates apoptosis induced by ciliary loss after KD of *KIF3A* or *IFT88*, we investigated whether inhibiting VDAC1 oligomerization blunts apoptosis. TPC1 or BCPAP cells were treated with an inhibitor of VDAC1 oligomerization (DIDS). Annexin V and PI staining revealed that apoptosis was markedly less evident in *IFT88*-deficient TPC1 or BCPAP treated with DIDS (late apoptosis of *IFT88*-deficient TPC1 = 5.3 ± 0.28%, *p* = *0.001* and early apoptosis of *IFT88*-deficient TPC1 = 2.3 ± 0.42%, *p* = *0.046*; late apoptosis of *IFT88*-deficient BCPAP = 14.9 ± 0.85%, *p* = *0.001* and early apoptosis of *IFT88*-deficient BCPAP = 9.6 ± 0.71%, *p* = *0.003*) than in cells not treated with DIDS (late and early apoptosis of *IFT88*-deficient TPC1 = 15.2 ± 0.71% and 3.4 ± 0.28%; late and early apoptosis of *IFT88*-deficient BCPAP = 27.2 ± 0.35% and 15.9 ± 0.14%) (Fig. 3G and H). Taken together, these results suggest a strong link between increased apoptosis after ciliary loss and VDAC1 oligomerization.

### Increased apoptosis of PTCs with ciliary loss is associated with reduced tumor aggressiveness

**Figure 4:**
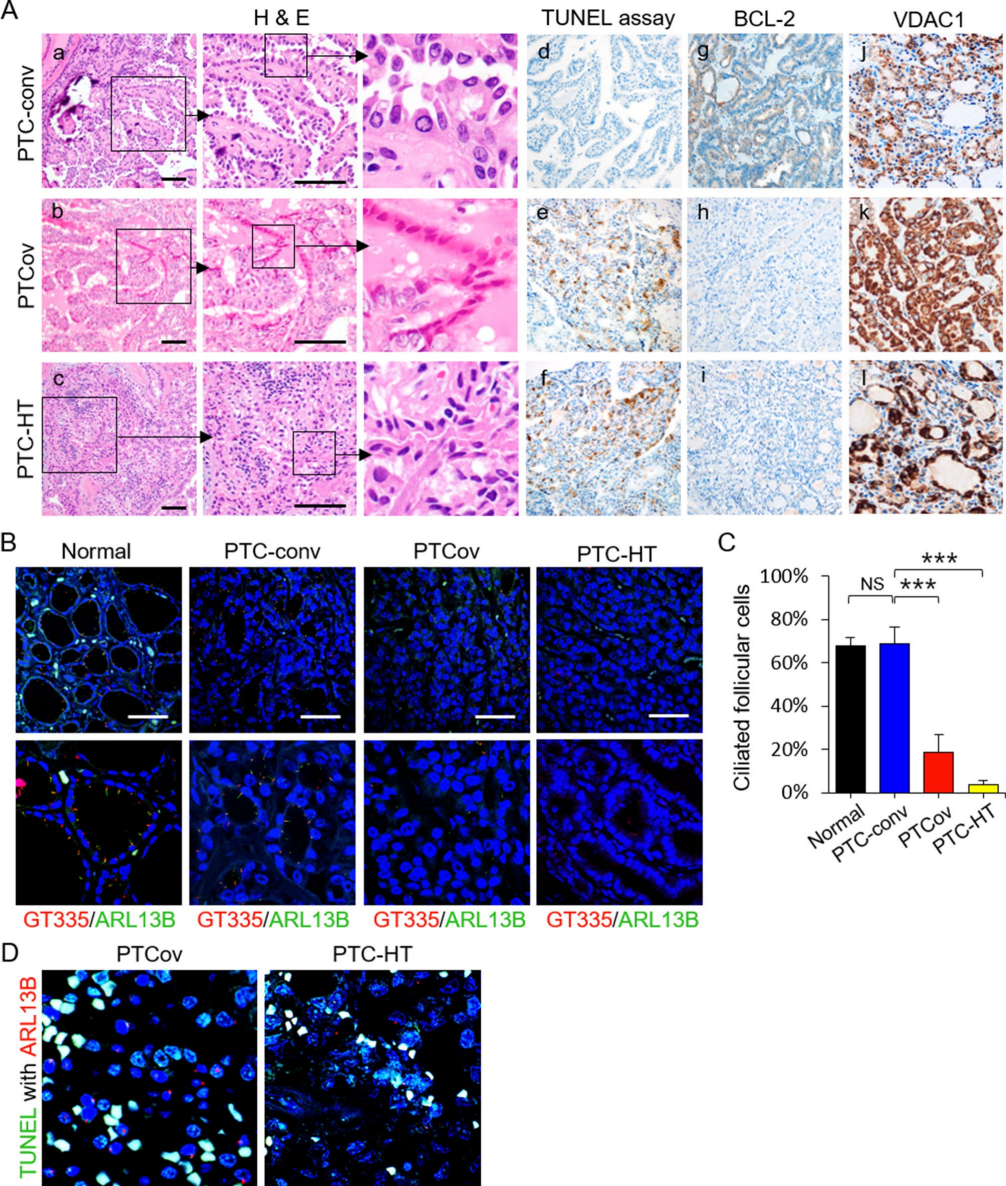
The proportion of apoptotic cancer cells increases in PTCs showing ciliary loss. (**A**) Upon histological examination after H & E staining, apoptotic cancer cells appeared dark, with an eosinophilic cytoplasm and dense purple nuclear chromatin fragments (insert). TUNEL analysis revealed significantly more apoptotic cancer cells in PTCov and PTC-HT tissues than in PTC-conv tissue (d, e, and f). BCL-2 expression levels were lower in PTCov and PTC-HT tissues than in PTC-conv tissue (g, h, and i). Immunoexpression of VDAC1 was higher in PTCov and PTC-HT than in PTC-conv (j, k, and l). Scale bar, 10 μm. (**B**, **C**) Immunofluorescence images show labeling of primary cilia in normal human thyroid follicles and PTCs by antibodies specific for GT335 and ARL13B. Fewer primary cilia were detected in oncocytic cancer cells in PTCov and PTC-HT tissue than in normal thyroid follicles or conventional PTC tissue. Scale bar, 10 μm. Abbreviations: PTC-conv, conventional papillary thyroid carcinoma; PTCov, oncocytic variant of PTC; PTC-HT, PTC with Hashimoto’s thyroiditis background. ***P < 0.001, NS; not significant. (**D**) TUNEL with ARL13B double immunofluorescence staining revealed that TUNEL-positive cancer cells (green) have no primary cilia (stained by ARL13B; red).

Apoptosis of thyroid follicular cells plays an important role in the pathogenesis of thyroid carcinoma and autoimmune thyroid disorders such as Hashimoto’s thyroiditis^18^. In fact, apoptotic cancer cells are often observed in PTCov and PTC-HT^18,19^. Likewise, we also confirmed that apoptotic cells with characteristic features, including cell shrinkage, dark eosinophilic cytoplasm, and dense shrunken pyknotic nuclei were more frequently observed in PTCov and PTC-HT (Fig. 4A-a–c). PTCov and PTC-HT tissue sections showed a marked increase in TUNEL-positive thyroid cancer cells (Fig. 4A-d–f), whereas expression of the anti-apoptotic protein BCL-2 was downregulated (Fig. 4A-g–i). Cancer cells in PTCov and PTC-HT tissue sections showed much higher expression of VDAC1 protein than conventional PTC (PTC-conv) (Fig. 4A-j–l).

Subsequently, we investigated the frequency of primary cilia in cancer cells from PTCov and PTC-HT. As expected, cancer cells from PTCov and PTC-HT rarely displayed primary cilia (Fig. 4B): normal thyroid follicles, 67.8 ± 3.6%; PTC-conv, 68.7 ± 7.8% versus PTCov 18.8 ± 7.9% (*p* < 0.0001); PTC-conv versus PTC-HT 3.6 ± 1.9% (*p* < 0.0001) (Fig. 4C). Furthermore, we performed a TUNEL assay with ARL13B double immunofluorescence staining to clearly establish the relationship between apoptosis and primary cilia in vivo. Compared with those on TUNEL-negative cancer cells, primary cilia in TUNEL-positive apoptotic cancer cells were barely detectable (Fig. 4D).

To investigate whether PTCs with apoptosis(+)/primary cilia (−) were associated with tumor behavior, we analyzed the relationship between apoptotic cancer cells lacking cilia and clinicopathological parameters (Tables 1 and 2). PTCov and PTC-HT lacking cilia were more closely associated with increased cancer cell apoptosis than PTC-conv. PTCs with apoptosis(+)/primary cilia(−) were inversely associated with extrathyroidal invasion (Table 2). Therefore, increased apoptosis of cancer cells in PTCs with ciliary loss is associated with indolent tumor behavior.

**Table 1:** Clinicopathologic characteristics of human thyroid cancer cases.

	PTC-conv	PTCov	PTC-HT
N (total = 80)	35	14	31
Age (years)	46.7 ± 12.8	50.4 ± 8.7	43.9 ± 10.3
**Sex**			
Male	0	0	0
Female	35	14	31
Tumor size (cm)	1.83 ± 1.03	1.90 ± 0.91	1.60 ± 0.89
**Apoptosis**			
Positive	4 (5.0%)**	12 (15.0%)*	31 (38.8%)**
Negative	31 (38.8%)**	2 (2.5%)*	0 (0.0%)**
**Extrathyroidal extension**			
Positive	24 (30.0%)	12 (15.1%)*	9 (11.3%)**
Negative	11 (13.8%)	2 (2.5%)*	22 (27.5%)**
**Multiplicity**			
Positive	9 (11.3%)	4 (5.0%)	6 (7.5%)
Negative	26 (32.5%)	10 (12.5%)	25 (31.3%)
**Bilaterality**			
Positive	7 (8.8%)	3 (3.8%)	5 (6.3%)
Negative	28 (35.0%)	11 (13.8%)	26 (32.5%)
**TNM stage**			
I	30 (37.5%)	9 (11.25%)	29 (36.25%)
II	5 (6.26%)	5 (6.25%)	2 (2.50%)
III	0 (0.0%)	0 (0.0%)	0 (0.0%)
IV	0 (0.0%)	0 (0.0%)	0 (0.0%)

Data represent the mean ± standard deviation. *P < 0.05, **P < 0.01.

*PTC-conv* conventional papillary thyroid carcinoma, *PTCov* oncocytic variant of PTC, *PTC-HT* PTC with Hashimoto’s thyroiditis.

**Table 2:** Association between apoptosis and clinicopathological characteristics of PTCs.

	Apoptosis with ciliary loss		*P* value
	Negative	Positive	
N (total = 80)	33	47	
Age at diagnosis (years)	45.1 ± 11.2	45.6 ± 9.7	0.415
**Histological subtype**			
PTC-conv	31 (38.8%)	4 (5.0%)	< 0.001
PTCov	2 (2.5%)	12 (15.0%)	0.024
PTC-HT	0 (0.0%)	31 (38.8%)	< 0.001
Tumor size (cm)	1.54 ± 0.99	1.48 ± 0.95	0.420
Multiplicity	10 (12.5%)	9 (11.3%)	0.306
Bilaterality	8 (10.0%)	7 (8.8%)	0.346
Extrathyroidal invasion	24 (30.1%)	21 (26.3%)	0.044
TNM stage			0.544
I	29 (36.3%)	39 (48.7%)	
II	4 (5.0%)	8 (10.0%)	
III	0 (0.0%)	0 (0.0%)	
IV	0 (0.0%)	0 (0.0%)	

Data represent the mean ± standard deviation.

*PTC-conv* conventional papillary thyroid carcinoma, *PTCov* oncocytic variant of PTC, *PTC-HT* PTC with Hashimoto’s thyroiditis.

### Extramitochondrial VDAC1 is localized in the basal body of primary cilia

**Figure 5:**
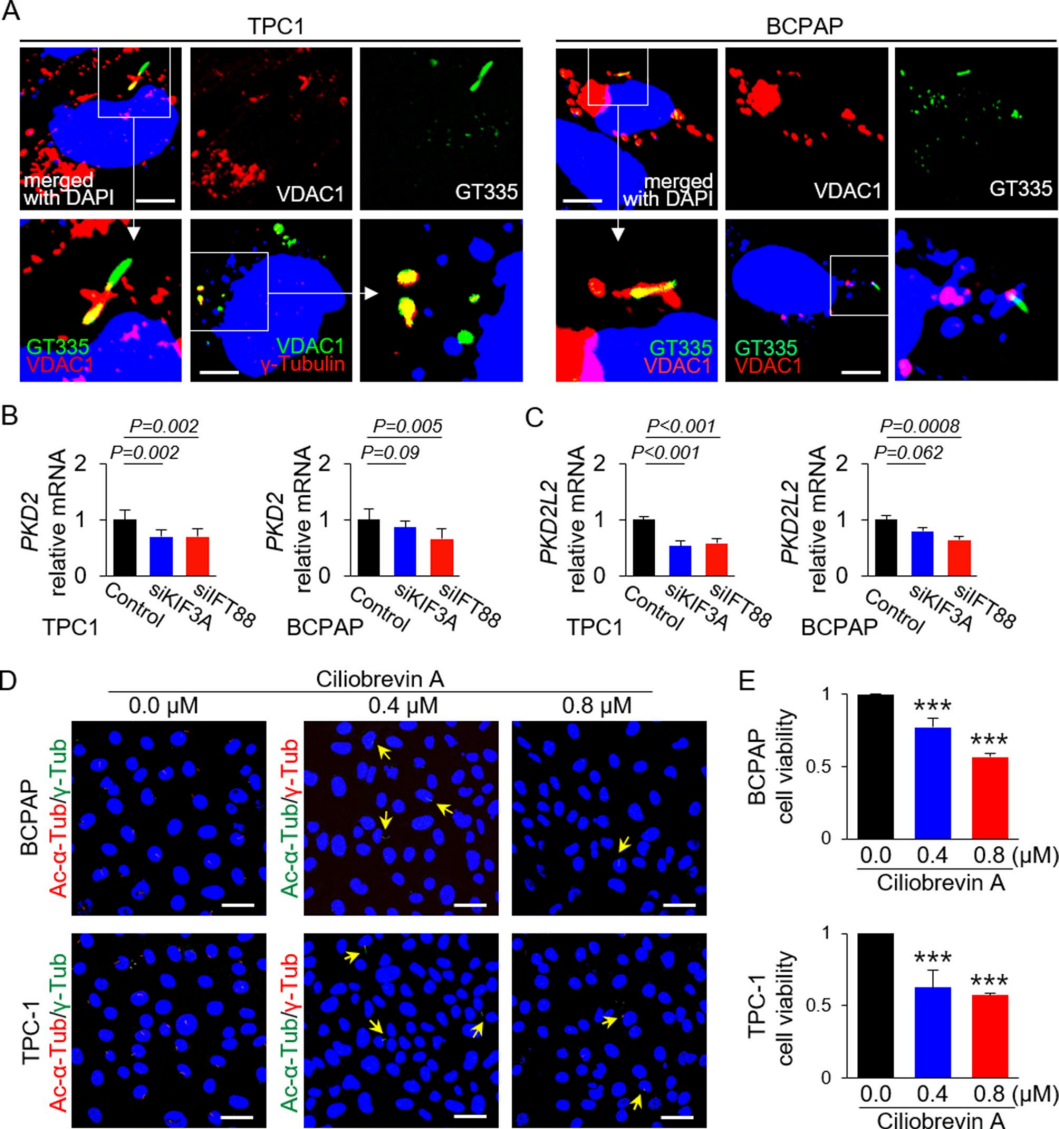
Extramitochondrial VDAC1 is localized in the basal body of primary cilia in PTC cells. (**A**) Immunofluorescence images show that VDAC1 localized to the basal body of primary cilia in thyroid cancer cell lines. Primary cilia were stained by anti-GT335 antibody (axonemes with basal bodies) and anti-γ-tubulin (basal bodies) antibodies. Scale bar, 10 μm. (**B**, **C**) Expression of *PKD2* and *PKD2L2* mRNA in *KIF3A*-deficient or *IFT88*-deficient thyroid cancer cell lines (TPC1 and BCPAP) compared with that in negative control siRNA-transfected cells. (**D**) BCPAP and TPC1 were incubated for 36 h with 0.4 μM or 0.8 μM ciliobrevin A. Immunofluorescence images show primary cilia stained with anti-acetylated-α-tubulin and anti-γ-tubulin antibodies. In the ciliobrevin A-treated groups, primary cilia are indicated by arrows. Scale bar, 10 μm. (**E**) The viability of BCPAP and TPC1 was evaluated in an EZ-cytox cell viability assay. Cell viability in the ciliobrevin A-treated groups was significantly lower than that in the untreated groups. ***P < 0.001.

During immunofluorescence analysis of VDAC1 and primary cilia in thyroid cancer cells, we found that extramitochondrial VDAC1 localized in the primary cilia. Therefore, we investigated the possible interactions between VDAC1 and primary cilia components using immunofluorescence analysis. VDAC1 co-localized with GT335-labeled primary cilia or γ-tubulin-labeled basal bodies in TPC1 and BCPAP cells (Fig. 5A). This result indicates that VDAC1 localizes to the basal body of primary cilia in thyroid cancer cells. This led us to hypothesize that VDAC1 expression in the basal body is connected to overexpression of VDAC in thyroid cancer cells showing LOF of primary cilia.

Primary cilia function as a specialized Ca^2+^-signaling comparent, and ciliary membranes contain several types of Ca^2+^ channel^20^. VDAC1 also possesses Ca^2+^-binding sites and forms the major Ca^2+^ ion-transport channel in the MOM. An increase in mitochondrial Ca^2+^ causes VDAC1 oligomerization, which then induces apoptosis by forming a large pore to enable passage of cytochrome *c*^21^. As with mitochondrial VDAC1, VDAC1 in the basal body may harbor Ca^2+^-binding sites and act as an intraciliary calcium signal messenger. Expression of mRNA encoding polycystin-2 (PKD2), a major ciliary Ca^2+^ channel in thyroid follicular cells, was lower in thyroid papillary carcinoma cells with LOF of primary cilia than in those with primary cilia (Fig. 5B). Expression of mRNA encoding polycystin 2-like 2 (PKD2L2), which functions to maintain high ciliary Ca^2+^ concentrations, was significantly downregulated in thyroid papillary carcinoma cells with LOF of primary cilia (Fig. 5C). This finding suggests that loss of ciliary function and structure results in decreased expression of calcium-regulating genes in primary cilia. The reduced calcium sensing caused by defective ciliogenesis may lead to increased (compensatory) expression and oligomerization of mitochondrial VDAC genes.

Next, we investigated whether pharmacological inhibition of ciliogenesis in thyroid cancer cells affects viability. Ciliobrevin A, a Hedgehog pathway antagonist, inhibits ciliogenesis^22^. After treatment with ciliobrevin A (0.4 µM or 0.8 µM), a few primary cilia were detected in thyroid cancer cells (Fig. 5D). The viability of ciliobrevin A-treated cells was significantly lower than that of untreated cells [BCPAP: 0 µM ciliobrevin A, 98.38 ± 2.87% versus 0.4 µM ciliobrevin A, 76.09 ± 5.96% (*p* < 0.0001) versus 0.8 µM ciliobrevin A, 55.84 ± 2.31% (*p* < 0.0001); TPC1: 0 µM ciliobrevin A, 100.54 ± 5.05% versus 0.4 µM ciliobrevin A, 62.98 ± 12.14% (*p* < 0.0001) versus 0.8 µM ciliobrevin A, 57.51 ± 1.42% (*p* < 0.0001)] (Fig. 5E). This means that drugs that inhibit ciliogenesis might form the basis of a new therapeutic strategy to target differentiated thyroid cancers.

## Discussion

Herein, we demonstrate the interplay between primary cilia and mitochondria-dependent apoptosis in differentiated thyroid cancer cells. Genetic defects in ciliogenesis and the resulting dysfunction of primary cilia in thyroid cancers led to marked upregulation of *VDAC1* genes and proteins, VDAC1 oligomerization, and apoptotic cell death. Thus, LOF of primary cilia in thyroid cancer cells acts as a novel apoptogenic stimulus that modulates the mitochondria-dependent apoptosis pathway. Furthermore, pharmacological suppression of ciliogenesis reduced the viability of thyroid cancer cells, suggesting a new therapeutic approach for differentiated thyroid cancers.

The primary cilium has a microtubule-based axoneme and a basal body. The basal body is modified from the mother centriole during quiescence or during G1 phase of the cell cycle, and serves as a nucleation site for assembly/disassembly of the axoneme microtubules^23^. In fact, upregulated ciliogenesis is inversely correlated with cell cycle progression^24,25^. Therefore, primary cilia play a crucial role at the point where the cell cycle pathway and the cell death pathway interact; thus primary cilia maintain the balance between cell cycle progression and apoptosis.

The primary cilia of thyroid cancer cells regulate bioenergetic metabolic reprogramming^26^ and provide a convergence point for cell cycle progression and apoptotic cell death. Primary cilia of renal tubular cells function to sense urine flow and osteocyte primary cilia are responsible for bone mechanotransduction^27,28^. Because primary cilia are inserted into the apical membrane of thyroid follicular cells, they can sense the follicular luminal environment. The sensory function of primary cilia in the thyroid follicle lumen may be lost in thyroid cancer cells because malignant thyroid follicular cells showing loss of polarity do not form organized thyroid follicles containing thyroglobulin^3^. Moreover, when primary cilia were removed from mouse thyroid follicles, thyroid cancer developed^3^. In this context, the primary cilia in thyroid cancer cells may be one of the important components that can be reprogrammed during cancer development. We showed previously that specific forms of thyroid cancer, such as Hürthle cell carcinoma, PTCov, and PTC-HT, show both reduced ciliogenesis and functional alterations in mitochondria^2,26^. Moreover, we showed previously that ATC cell lines (SW1736, Hth7) have a lower ciliated frequency^3^ and genetic loss of primary cilia in thyroid gland affects tumorigenesis and progression of thyroid cancer^26^. In *Tg-Cre;Ift88*^flox/flox^ mice, the thyroids of young mice are composed of irregularly dilated follicles formed via apopotosis, which develop into proliferative solid nodules with age. Taken together, these results suggest that loss of primary cilia may play a role in the selection of a subpopulation of thyroid cancer cells with more malignant features.

Here, we demonstrate that marked reductions in ciliogenesis are linked to mitochondria-dependent apoptosis via modulation of VDAC1. We also show that VDAC1 is present in the basal body of primary cilia. By exploring functional interactions between VDAC1 in basal bodies and mitochondria under conditions of impaired ciliogenesis, we show that VDAC1 located in the basal body may play a role in communication with mitochondria. The function of VDAC1 in the basal body VDAC1 remains unclear, and its role in regulating mitochondrial VDAC in thyroid cancer cells needs to be verified. We found that defective *KIF3A*-mediated or defective *IFT88*-mediated ciliary loss results in reduced expression of PKD2 and PKD2L2, which control calcium homeostasis. Based on these results, we propose that reduced calcium sensing caused by defective ciliogenesis may mediate increased (compensatory) expression and oligomerization of mitochondrial VDAC.

Ciliogenesis, particularly inhibition of ciliogenesis, is a therapeutic target for cancer^29,30^. Small molecules that inhibit ciliogenesis display anticancer activity^31^, although the mode of action remains unclear. Here, we demonstrate a sequential process of cell death in thyroid cancer cells with LOF of primary cilia; this process is characterized by VDAC1 oligomerization, cytochrome c release/increase in intracellular Ca^2+^ levels, and induction of apoptosis. In fact, anticancer drugs such as cisplatin, arbutin, somatostatin, and prednisolone exert anti-tumor activity by regulating VDAC1^32–35^. In addition, many compounds that induce apoptosis in cancer cells by modulating VDAC are already being tested in clinical trials^36^. Much research is being undertaken to find new therapeutic targets that modulate VDAC activity. Therefore, regulation of VDAC1 by ciliogenesis inhibitors or regulators might be a therapy for thyroid cancers. To the best of our knowledge, this is the first study to demonstrate that LOF of primary cilia in PTCs acts as an apoptogenic stimulus that modulates the mitochondria-dependent apoptosis pathway.

In conclusion, we show that LOF of primary cilia in differentiated thyroid cancer cells increases VDAC1 oligomerization and induces mitochondria-dependent apoptosis. The results provide evidence that drugs that induce thyroid cancer cell-specific ciliary loss have potential as new therapeutics for differentiated thyroid cancer.

## Materials and methods

### Mice

Floxed Ift88 (Ift88^flox/flox^) mice and thyroglobulin-cre (Tg-Cre) mice were obtained from Dr. Kim J (Korea Advanced Institute of Science and Technology, Daejeon, Korea) and Dr. Jukka Kero, respectively. These mice were on a C57BL/6 genetic background. Ift88^flox/flox^ mice were crossed with Tg-Cre transgenic mice to generate thyroid follicular cell-specific Ift88-knockout (*Tg-Cre;Ift88*^*flox/flox*^) mice. All animal experiments received prior approval by the Institutional Animal Care and Use Committee of the Catholic University of Korea (approval ID, CRCC-BE-CMC-17013391) and were performed in accordance with the guidelines and regulations of the Catholic University of Korea.

The presence of primary cilia in the thyroid gland of adult C57BL/6 J mice was confirmed by immunofluorescence analysis. After removing the parathyroid gland using a stereo microscope, only thyroid tissue was excised. The extracted thyroid was divided in half using a surgical blade (No. 11) and the cut surface was smeared onto a glass slide. The smear slide was fixed for 20 min in 4% paraformaldehyde in PBS, followed by immunofluorescence staining. The primary antibodies used were specific for acetylated α-tubulin or ARL13B (axoneme), and γ-tubulin (basal body). The axoneme of murine thyroid follicular cells was stained weakly by the anti-ARL13B antibody, but stained intensely by the acetylated α-tubulin antibody. Interestingly, the primary cilia of murine thyroid follicular cells had a short axoneme that was almost the same size as the basal body (Supplementary Fig. S3). Based on these cytology findings, it was possible to confirm the presence of primary cilia in *Tg-Cre;Ift88* floxed mice (Fig. 1A).

### Human papillary thyroid cancer tissues

Formalin fixation and paraffin embedded (FFPE) tissue blocks of human thyroid (normal, conventional papillary thyroid carcinoma [PTC] oncocytic variant of PTC [PTCov], PTC with Hashimoto’s thyroiditis [PTC-HT]) were obtained from patients that underwent a thyroidectomy between January 2002 and December 2005 at St. Mary’s Hospital, Daejeon, South Korea. All participants gave informed consent. The American Joint Committee on Cancer (AJCC, 8th edition) TNM classification system was used for the thyroid cancer staging. Furthermore, we retrospectively followed the patient medical records to evaluate tumor recurrence. A 10-year follow-up did not reveal any local recurrences or distant metastases of thyroid cancer. The study protocol was reviewed and approved by the Institutional Review Board of Daejeon St. Mary's Hospital (approval ID, DC20SISI0056) and all methods were carried out in accordance with the guidelines of Daejeon St. Mary's Hospital.

### Culture of human thyroid cancer cell lines

TPC1 (a human RET/PTC rearrangement PTC cell line; from Dr. Takahashi M, Nagoya University, Japan) and BCPAP (a human BRAF^V600E^ mutant PTC cell line; from Dr. M. Santoro, Università di Napoli Federico II, Italy) were cultured in RPMI 1640 (WELGENE Inc. Republic of Korea) and Dulbecco’s Modified Eagle’s Medium (DMEM, WELGENE Inc. Republic of Korea), respectively, supplemented with 10% fetal bovine serum (FBS, HyClone USA) and 1% penicillin/streptomycin at 37 °C/5% CO_2_.

### Generation of thyroid cancer cell lines in which KIF3A or IFT88 were knocked down

Cells were plated in 30-mm tissue culture dishes 24 h prior to transfection such that cells were 60% confluent at the time of transfection. TPC1 and BCPAP cells were transfected with 20 nM siRNA specific for *KIF3A* (si*KIF3A*) or IFT88 (si*IFT88*) (Invitrogen) in Opti-MEM I using Lipofectamine RNAiMAX transfection reagent (Invitrogen), according to the manufacturer’s optimized protocols. Negative control siRNAs containing non-specific sequences with no homologs in the human genome were also provided by Invitrogen. All cells were used at 48 h post-transfection. All experiments were performed in triplicate and repeated at least three times. KD efficiency was determined by quantitative RT-PCR.

### TUNEL assay to detect and quantitate apoptotic cells

To investigate apoptotic cell death, we measured apoptosis in each tissue slide using a TUNEL Assay Kit-HRP-DAB (Abcam), according to the manufacturer's instructions.

The DeadEnd Fluorometric TUNEL System (Promega) was used to conduct TUNEL with double immunofluorescence staining. The anti-goat ARL13B primary antibody specific for primary cilia was detected using an anti-rabbit IgG secondary antibody conjugated to Alexa Fluor 594 (red fluorescence). This technique was used to detect primary cilia in apoptotic thyroid follicular cells (co-labeled with green and red fluorescence).

### Immunohistochemistry

FFPE tissue blocks were cut into sections (4-μm thick) and the slides were incubated in an oven at 56 °C for 30 min. The immunohistochemical assay was performed using the Ventana HX automatic BenchMark system (Ventana Medical Systems, SA, Illkirch Cedex, France). The primary antibodies were anti-mouse Bcl-2 (BD Pharmingen) and anti-rabbit VAC1 (Abcam). Slides were cover slipped and analyzed under an OLYMPUS BX51 microscope.

### Immunofluorescence staining

Cells were plated on round coverslips in 12-well plates. After incubation under each experimental condition, cells were washed with 1 × PBS and fixed for 20 min at room temperature with 4% paraformaldehyde in PBS. After washing three times with 1 × PBS (10 min each), cells were permeabilized for 10 min with 0.5% Triton X-100 in PBS. After washing three times with 1 × PBS (10 min each time), cells were blocked for 30 min at room temperature with 3% bovine serum albumin in PBS. Thereafter, cells were incubated overnight at 4 °C with primary antibodies specific for acetylated α-tubulin (Cell Signaling Technology), polyglutamylation modification (GT335, AdipoGen), ARL13B (ProteinTech Group), γ-tubulin (Sigma-Aldrich), and VDAC1 (Abcam). After washing three times with 1 × PBS (10 min each time), cells were incubated for 3 h at room temperature with secondary antibodies (goat anti-mouse and goat anti-rabbit secondary antibodies conjugated to Alexa Fluor dyes (Invitrogen/Life Technologies)). Nuclei were stained with DAPI. After carefully removing the coverslips from the wells, the coverslips were mounted with the cells facing towards the microscope slide. The stained slides were observed under an Olympus FluoView FV1000 microscope equipped with a charge-coupled device.

To observe the mitochondrial networks, MitoTracker Red (Invitrogen) was incubated with cultured live cells for 20 min prior to paraformaldehyde fixation. To stain tissue sections, FFPE tissue blocks were sectioned (7-μm thick), deparaffinized, and heated to 121 °C for 25 min in citrate buffer prior to antigen retrieval. After treatment with 0.5% Triton X-100, the procedure was the same as that for the aforementioned cell staining method.

### Preparation of mitochondrial and cytosol fractions of tumor cells

Negative control siRNA-transfected TPC1 and BCPAP, *KIF3A*-KD TPC1 and BCPAP, and *IFT88*-KD TPC1 and BCPAP cells were grown in 100-mm dishes. After transfection for 48 h, the cells were washed with 1 × PBS and harvested using 0.05% Trypsin–EDTA solution (Gibco). Cells were centrifuged at 1000 rpm for 10 min at 4 °C. The supernatant was discarded from the conical tube and the cell pellet was suspended in isolation buffer [210 mM Mannitol, 70 mM Sucrose, 1 mM EGTA, 5 mM Tris–Cl (pH 7.5)] containing a protease inhibitor (cOmplete ULTRA Tablets, Roche). Cells were homogenized on ice using a Teflon-glass Potter–Elvehjem homogenizer and then centrifuged at 600 × g for 10 min at 4 °C. The supernatant was recentrifuged at 17,000 × g for 10 min at 4 °C and the final supernatant was used as the cytosolic fraction. The pellet was resuspended in RIPA lysis buffer containing protease inhibitor and used as the mitochondrial fraction.

### SDS-PAGE and western blot analyses

Cells were washed twice with cold PBS and lysed in RIPA lysis buffer (10 mM Tris–HCl pH 8.0, 150 mM NaCl, and 1% Nonidet P-40) supplemented with a protease inhibitor. Protein concentrations were measured using the Bradford assay and protein samples were prepared by addition of SDS sample buffer. Samples were denatured by boiling for 5 min, loaded onto a polyacrylamide gel, and separated by gel electrophoresis. The separated proteins were transferred to a nitrocellulose membrane using the wet transfer method. The membrane was incubated with blocking buffer for 30 min and then incubated overnight at 4 °C with appropriate primary antibodies specific for VDAC1 (Abcam), HSP60 (Santa Cruz Biotechnology), and GAPDH (Abcam). After the membrane was rinsed to remove unbound primary antibody, it was exposed to a secondary antibody for 2 h at room temperature. After washing three times in TBS/T (10 min each), the blot was developed using a chemiluminescent detection kit (Immobilon Western Chemiluminescent HRP Substrate, Merck Millipore).

### Total RNA isolation and RT-qPCR

The Easy-BLUE Total RNA Extraction Kit (iNtRON Bio) was used to extract total RNA from cultured cells. M-MLV Reverse Transcriptase and oligo-dT primers (Invitrogen) were used to synthesize complementary DNA (cDNA) from total RNA. RT-qPCR was performed using an Applied Biosystems 7500 Real-Time PCR System (Thermo Fisher scientific, USA) and QuantiTect SYBR Green PCR Master Mix (QIAGEN). Each reaction was carried out in triplicate. The sequences of the primers used for qPCR were as follows: *IFT88*-Forward (F) 5′-TGCAAAACTCATTGCTCCTG-3′ and *IFT88*-Reverse (R) 5′-CACGCACCAATCATAACCTG-3′; *KIF3A*-F 5′-CTCGTCTTCTTCAGGATTCC-3′, and *KIF3A*-R 5′-GAGACTTTCTTTTTTCCCCTTC-3′; *VDAC1*-F 5′-AAGTGAACAACTCCAGCCTGA-3′, and *VDAC1*-R 5′-CACCAGCATTGACGTTCTTG-3′; *VDAC2*-F 5′-CATTTCTGCAAAAGTCAACAACTC-3′ and *VDAC2*-R 5′-TCCCATCTACCAGAGCAGAGA-3′; *VDAC3*-F 5′-AGCCTGATTGGACTGGGTTA-3′ and *VDAC3*-R 5′-CTTGTGACCTCCTGCACTGA-3′; *PKD2*-F 5′-CCTAGCGTATGCTCAGTTGG-3′ and *PKD2*-R 5′-GTAGCCCTTTCTGATAAGATCTGAG-3′; *PKD2L2*-F 5′-GCGGAGATTTGGCTGAACAAGC-3′ and *PKD2L2*-R 5′-CTTGAGTGACAGGCTGGTAGTC-3′; *GAPDH*-F 5′-TGCCTCCTGCACCACCAACT-3′ and *GAPDH*-R 5′-ACACGTTGGCAGTGGGGACA-3’.

### Analysis of apoptosis using Annexin V-FITC and PI

Apoptosis induced by ciliary loss was assayed using Annexin V–fluorescein isothiocyanate (FITC) and propidium iodide (PI) staining, followed by fluorescence-activated cell sorting (FACS) analysis. Cells were plated in 6-well plates and transfected with 5 nM KIF3A siRNA or IFT88 siRNA for 48 h, after which the culture medium was exchanged for serum-free medium for 24 h. Cells were harvested and centrifuged at 300 × g for 5 min at room temperature. The supernatant was discarded and the cells were resuspended in culture medium at a concentration of 1 × 10^6^ cells/ml. The cells were then washed with FACS buffer and stained for 20 min with 5 μl of FITC-conjugated Annexin V (BD Pharmingen) and 5 μM PI (Sigma-Aldrich Inc.) prior to analysis using a FACS Canto-II flow cytometer (BD Biosciences).

To examine apoptosis after inhibiting VDAC1 oligomerization in thyroid cancer cell lines with ciliary loss, cells transfected for 48 h with siRNA were treated for a further 24 h with an inhibitor of VDAC1 oligomerization (4,4′-diisothiocyanostilbene-2,2′-disulfonic acid (DIDS); Sigma-Aldrich).

### Cell viability assay

Apoptotic cell death of human thyroid cancer cell lines was assessed using the LDH assay (Thermo Fisher Scientific Inc.). Cell viability after ciliary loss in thyroid cancer cell lines was evaluated using the MTT cell proliferation colorimetric assay (BioVision, Inc., K301). The absorbance value at 570 nm was read by an automatic multiwell spectrophotometer (Molecular Devices, Sunnyvale, CA, USA).

Additionally, cell viability was evaluated using the EZ-cytox Cell Viability Assay Kit (DoGenBio Co.). Briefly, thyroid cancer cells were seeded in 96-well plates (1 × 10^6^ cells/well) and cultured for 24 h. Next, cells were treated with ciliobrevin A (4 µM or 8 µM, Sigma-Aldrich) in serum free medium. After 36 h, cells were exposed to 10 µl of WST solution. Absorbance was measured 0, 60, and 90 min later in a plate reader at a wavelength of 450 nm (Molecular Devices, Sunnyvale, CA, USA).

### Statistical analysis

Statistical analyses were performed using the Chi-square test (IBM SPSS Statistics 22.0). Data are presented as the mean ± standard deviation (SD). The statistical significance of the differences between two groups was determined using Student’s t-test. A *P*-value < 0.05 was considered significant.

## Supplementary Information

Supplementary Information.

Supplementary Figures 1.

Supplementary Figures 2.
