# Supplementary material for: Loss of primary cilia promotes mitochondria-dependent apoptosis in thyroid cancer
Source: Sci Rep. 2021 Feb 18;11:4181. doi: 10.1038/s41598-021-83418-3 (PMC7893175; doi:10.1038/s41598-021-83418-3)
Supplement: Supplementary file 1 — Supplementary Information. [file 41598_2021_83418_MOESM1_ESM.docx]

**Supplementary Information**

**Loss of primary cilia promotes mitochondria-dependent apoptosis in thyroid cancer**

Junguee Lee^1^*, Ki Cheol Park^2^, Hae Joung Sul^1^, Hyun Jung Hong^3^, Kun-Ho Kim^4^, Jukka Kero^5^, Minho Shong^6^*

^1^Department of Pathology, Daejeon St. Mary’s Hospital, College of Medicine, The Catholic University of Korea, Seoul 06591, Republic of Korea.

^2^Clinical Research Institute, Daejeon St. Mary’s Hospital, College of Medicine, The Catholic University of Korea, Daejeon 34943, Republic of Korea.

^3^Research Center for Endocrine and Metabolic Diseases, Chungnam National University School of Medicine, Daejeon 35015, Republic of Korea.

^4^Department of Nuclear Medicine, Chungnam National University Hospital and College of Medicine, Daejeon 35015, Republic of Korea.

^5^Research Centre for Integrative Physiology and Pharmacology, Institute of Biomedicine, University of Turku, Kiinamyllynkatu 10, 20520 Turku, Finland.

^6^Department of Internal Medicine, Chungnam National University School of Medicine, 266 Munhwaro, Daejeon 35015, Republic of Korea.

*Correspondance; JL ([junguee@catholic.ac.kr](mailto:junguee@catholic.ac.kr)), MS ([minhos@cnu.ac.kr](mailto:minhos@cnu.ac.kr))

**SUPPLEMENTARY FIGURE LEGENDS**

**Supplementary Figure S1.** The cell selection process used in various experiments. (A) The LDH level, an indicator of apoptotic cell death, is lower in differentiated thyroid cancer cell lines, TPC1 and BCPAP, than in poorly differentiated thyroid cancer cell lines. (B) Frequency of primary cilia in thyroid carcinoma cell lines. (C) MTT analysis evaluating the viability of cells undergoing loss of primary cilia.

**Supplementary Figure S2.** Thyroid cancer cell lines lacking primary cilia were generated by knocking down KIF3A or IFT88. (A) RT-qPCR analysis of the knockdown (KD) efficiency of KIF3A and IFT88 in TPC1 cells (data are normalized to KIF3A or IFT88 expression by negative control siRNA-transfected cells). (B) RT-qPCR analysis of the KD efficiency of KIF3A and IFT88 in BCPAP cells (data are normalized to KIF3A or IFT88 expression by negative control siRNA-transfected cells).

**Supplementary Figure S3.** The presence of primary cilia in the thyroid gland of adult C57BL/6J mice. Methods used to make cell smears and subsequent immunofluorescence analysis. Primary cilia were confirmed by staining with anti-acetylated α-tubulin (Ac-α-Tub, green) and anti-γ-tubulin (γ-Tub, red) antibodies. The primary cilia of murine thyroid follicular cells have a short axoneme (Ac-α-Tub, green) that was almost the same size as the basal body (γ-Tub, red).
