# Supplementary material for: Loss of primary cilia promotes mitochondria-dependent apoptosis in thyroid cancer
Source: Sci Rep. 2021 Feb 18;11:4181. doi: 10.1038/s41598-021-83418-3 (PMC7893175; doi:10.1038/s41598-021-83418-3)
Supplement: Supplementary file 2 — Supplementary Figures 1. [file 41598_2021_83418_MOESM2_ESM.pptx]

## Slide 1
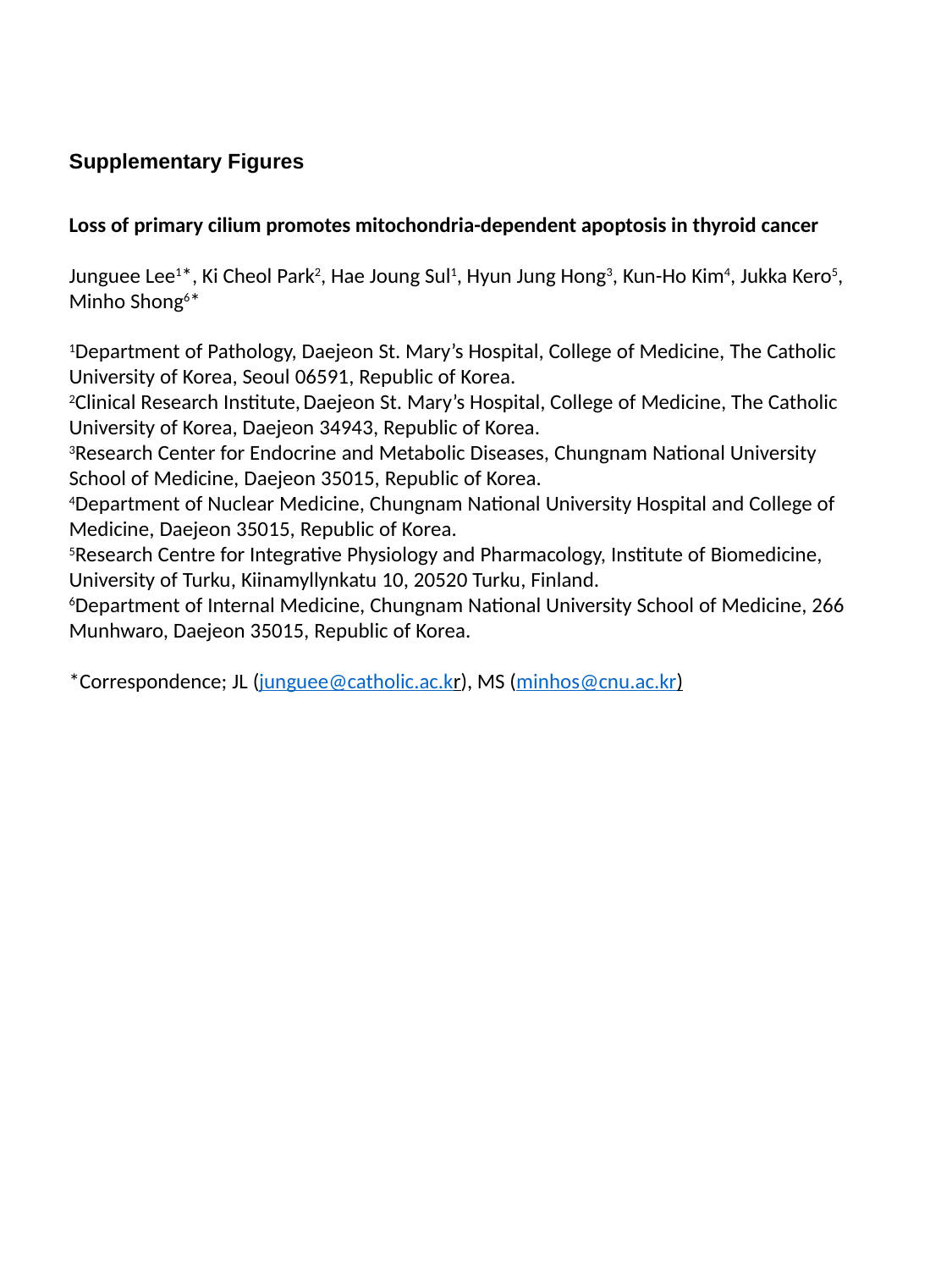

Supplementary Figures
Loss of primary cilium promotes mitochondria-dependent apoptosis in thyroid cancer
Junguee Lee1*, Ki Cheol Park2, Hae Joung Sul1, Hyun Jung Hong3, Kun-Ho Kim4, Jukka Kero5, Minho Shong6*
1Department of Pathology, Daejeon St. Mary’s Hospital, College of Medicine, The Catholic University of Korea, Seoul 06591, Republic of Korea.
2Clinical Research Institute, Daejeon St. Mary’s Hospital, College of Medicine, The Catholic University of Korea, Daejeon 34943, Republic of Korea.
3Research Center for Endocrine and Metabolic Diseases, Chungnam National University School of Medicine, Daejeon 35015, Republic of Korea.
4Department of Nuclear Medicine, Chungnam National University Hospital and College of Medicine, Daejeon 35015, Republic of Korea.
5Research Centre for Integrative Physiology and Pharmacology, Institute of Biomedicine, University of Turku, Kiinamyllynkatu 10, 20520 Turku, Finland.
6Department of Internal Medicine, Chungnam National University School of Medicine, 266 Munhwaro, Daejeon 35015, Republic of Korea.
*Correspondence; JL (junguee@catholic.ac.kr), MS (minhos@cnu.ac.kr)

## Slide 2
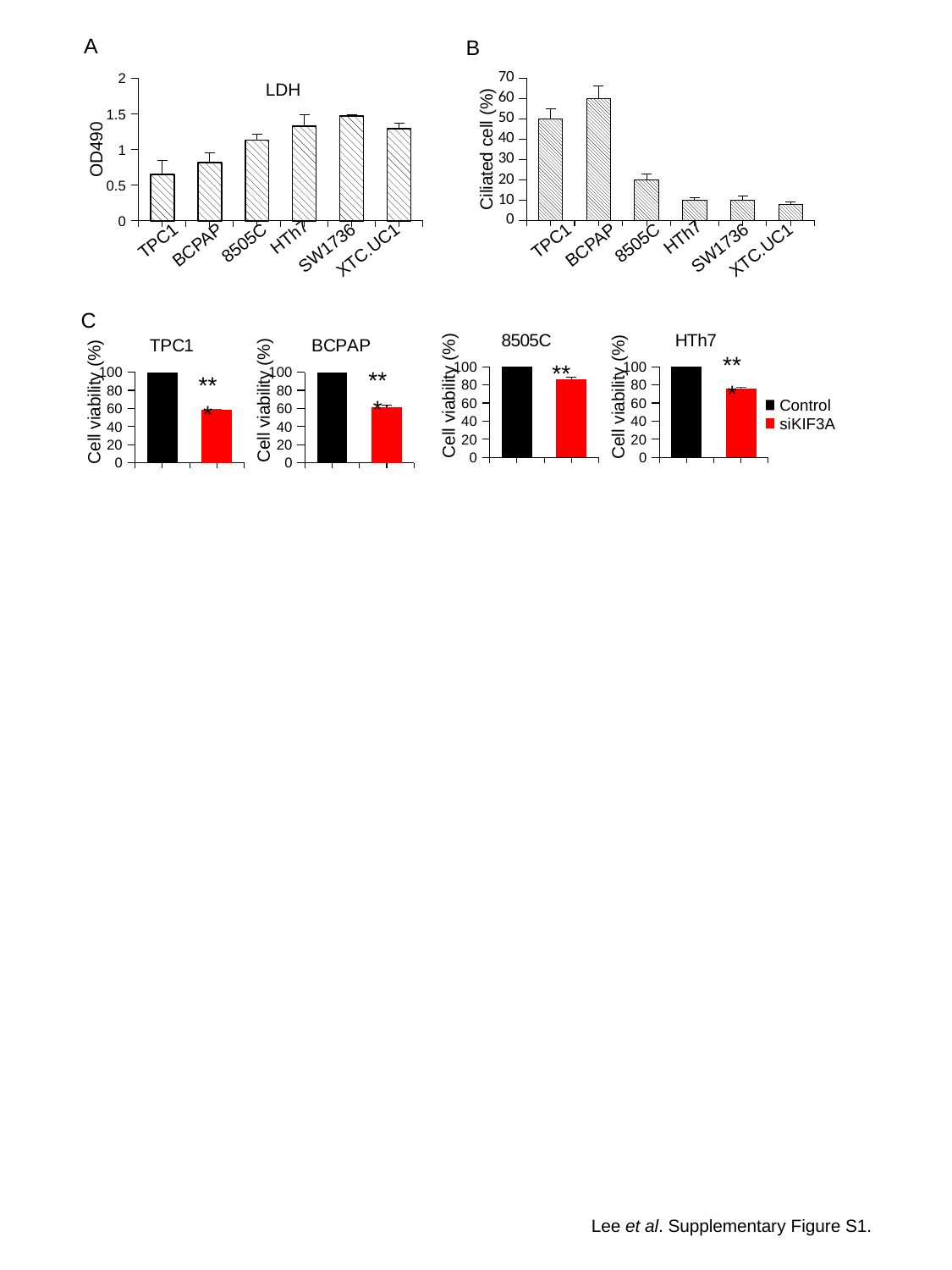

A
### Chart
| Category | |
|---|---|LDH
OD490
HTh7
TPC1
BCPAP
8505C
SW1736
XTC.UC1
B
### Chart
| Category | |
|---|---|Ciliated cell (%)
HTh7
TPC1
BCPAP
8505C
SW1736
XTC.UC1
C
### Chart: BCPAP
| Category | h7h83 |
|---|---|
| wt | 100.0 |
| kif3 ko | 61.43454486973303 |***
Cell viability (%)
### Chart: TPC1
| Category | |
|---|---|***
Cell viability (%)
### Chart: 8505C
| Category | bcpap |
|---|---|
| wt | 100.0 |
| kif3 ko | 86.51019440493126 |**
Cell viability (%)
### Chart: HTh7
| Category | |
|---|---|***
Cell viability (%)
Control
siKIF3A
Lee et al. Supplementary Figure S1.

## Slide 3
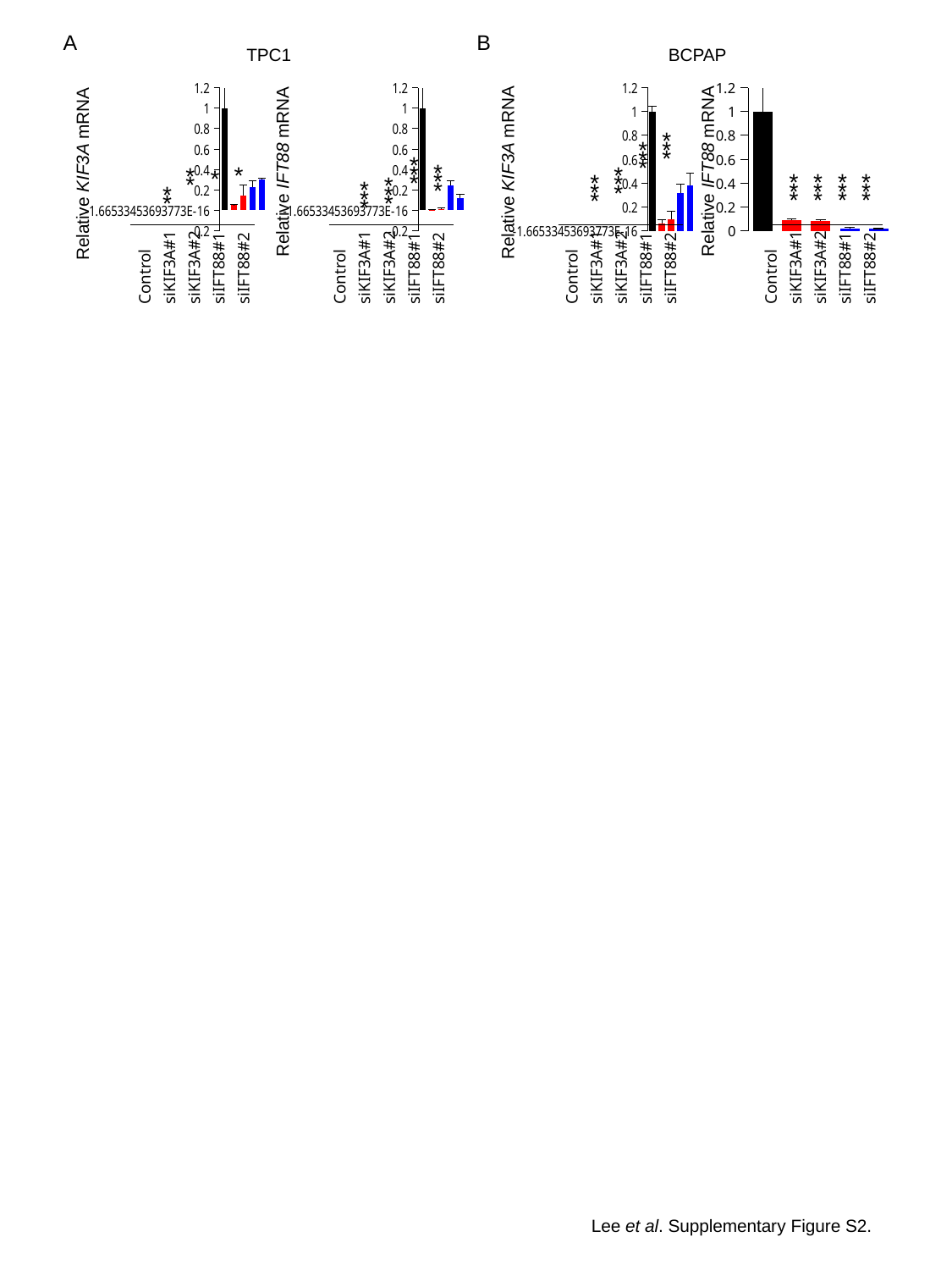

A
B
BCPAP
TPC1
### Chart
| Category | |
|---|---|***
***
Relative KIF3A mRNA
***
***
Control
siKIF3A#1
siKIF3A#2
siIFT88#1
siIFT88#2
### Chart
| Category | |
|---|---|Relative KIF3A mRNA
*
**
*
**
Control
siKIF3A#1
siKIF3A#2
siIFT88#1
siIFT88#2
### Chart
| Category | |
|---|---|Relative IFT88 mRNA
***
***
***
***
Control
siKIF3A#1
siKIF3A#2
siIFT88#1
siIFT88#2
### Chart
| Category | |
|---|---|Relative IFT88 mRNA
***
***
***
***
Control
siKIF3A#1
siKIF3A#2
siIFT88#1
siIFT88#2
Lee et al. Supplementary Figure S2.

## Slide 4
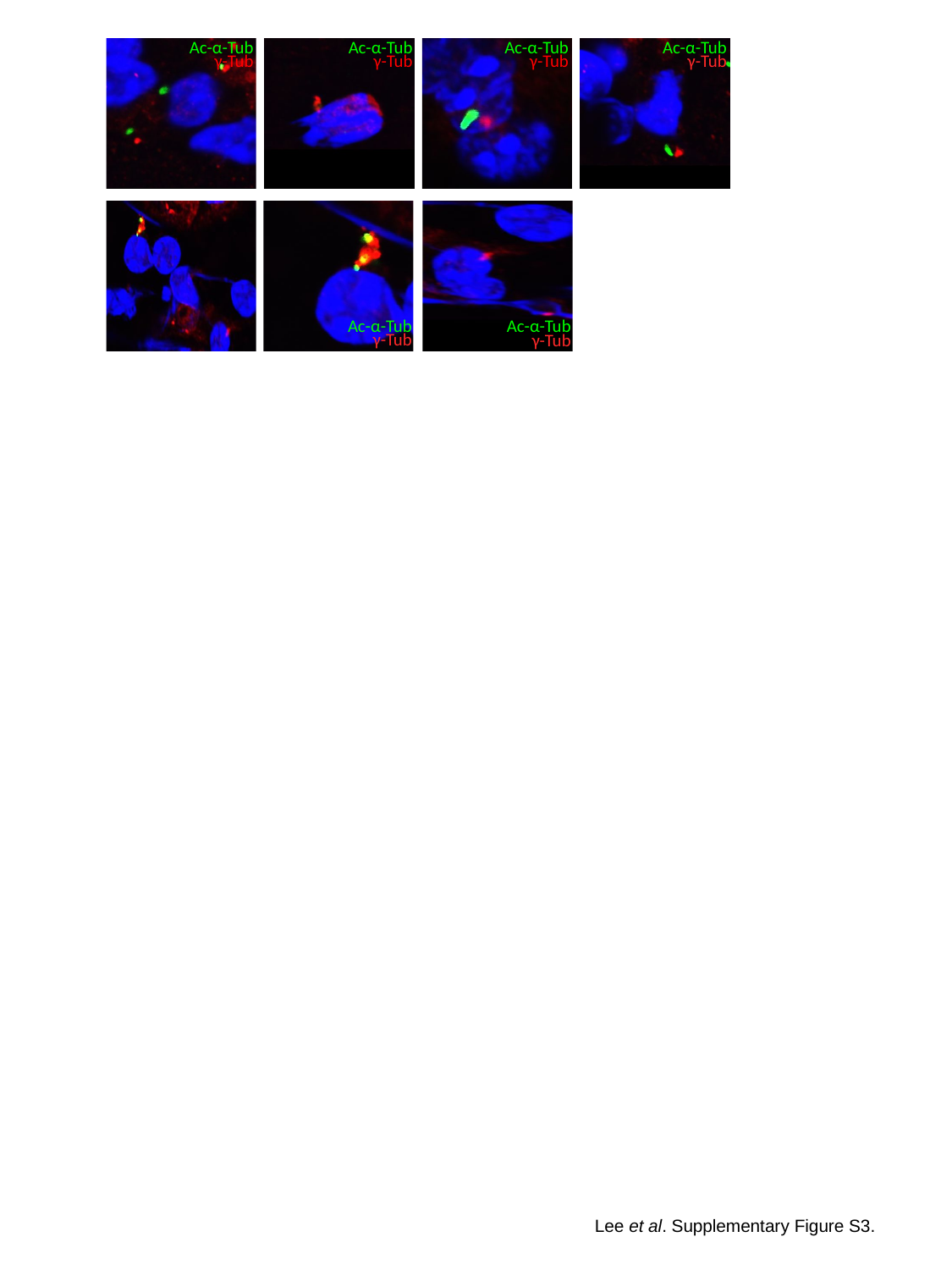

Ac-α-Tub
γ-Tub
Ac-α-Tub
γ-Tub
Ac-α-Tub
γ-Tub
Ac-α-Tub
γ-Tub
Ac-α-Tub
γ-Tub
Ac-α-Tub
γ-Tub
Lee et al. Supplementary Figure S3.
