# Supplementary material for: Loss of primary cilia promotes mitochondria-dependent apoptosis in thyroid cancer
Source: Sci Rep. 2021 Feb 18;11:4181. doi: 10.1038/s41598-021-83418-3 (PMC7893175; doi:10.1038/s41598-021-83418-3)
Supplement: Supplementary file 3 — Supplementary Figures 2. [file 41598_2021_83418_MOESM3_ESM.pptx]

## Slide 1
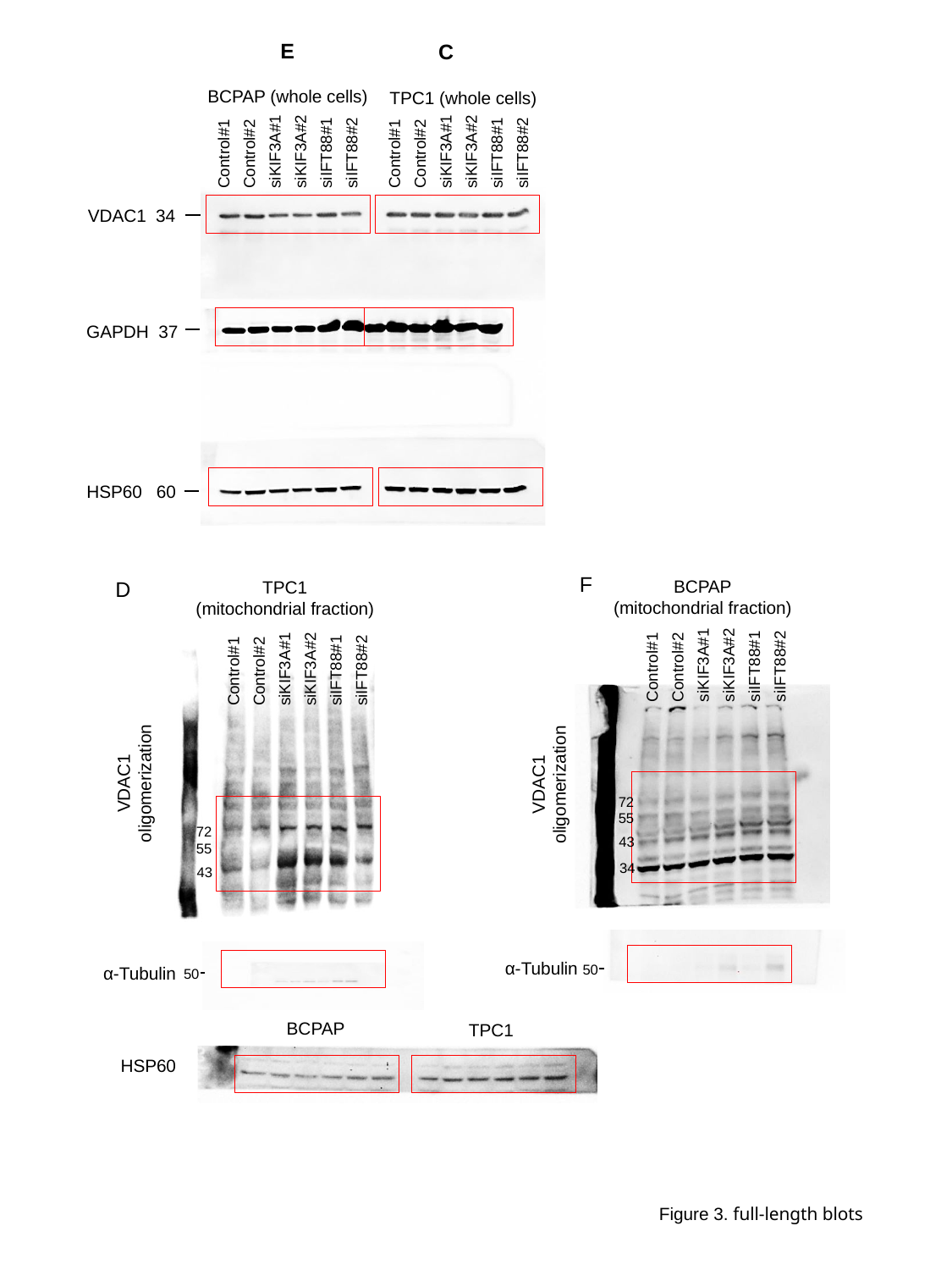

E
C
Control#1
Control#2
siKIF3A#1
siKIF3A#2
siIFT88#1
siIFT88#2
Control#1
Control#2
siKIF3A#1
siKIF3A#2
siIFT88#1
siIFT88#2
BCPAP (whole cells)
TPC1 (whole cells)
VDAC1 34
GAPDH 37
HSP60 60
F
BCPAP
(mitochondrial fraction)
D
TPC1
(mitochondrial fraction)
Control#1
Control#2
siKIF3A#1
siKIF3A#2
siIFT88#1
siIFT88#2
Control#1
Control#2
siKIF3A#1
siKIF3A#2
siIFT88#1
siIFT88#2
VDAC1 oligomerization
VDAC1 oligomerization
72
55
72
43
55
34
43
α-Tubulin
α-Tubulin
50
50
BCPAP
TPC1
HSP60
Figure 3. full-length blots
